# Rapamycin increases breast tumor burden in young wheel-running mice

**DOI:** 10.1080/20010001.2019.1647746

**Published:** 2019-07-26

**Authors:** Juan Wang, Thomas Truong, Warren Ladiges, Jorming Goh

**Affiliations:** a Department of Comparative Medicine, University of Washington, Seattle, WA, USA; b Interdisciplinary Program in Nutritional Sciences, University of Washington, Seattle, WA, USA

**Keywords:** Rapamycin, wheel running, aging intervention, mouse model, breast cancer, age-related disease

## Abstract

Rapamycin is an immunosuppressive and anti-cancer drug recently shown to enhance healthy aging in animal models. Regular physical exercise is associated with healthy aging and reduced risk of age-related diseases, such as cancer. In order to test the combined effect of these approaches, mice with 4T1 breast cancer were fed rapamycin at 14 ppm and allowed access to voluntary running wheels. After 17 days of treatment, mice fed the rapamycin diet that ran showed a significant increase in tumor burden compared with mice that did not run (P = 0.017). Not only does this have implications for young breast cancer patients, but suggests that combining rapamycin and exercise as an anti-aging strategy at a young age might be contraindicated.

Rapamycin was originally developed as an immunosuppressive drug for organ transplantation [1]. It has also been used clinically for various types of cancers including breast cancer [3], and recently has been shown to enhance healthy aging in animal models [4,5]. In this regard it is of interest to see if additional intervention approaches can be combined with rapamycin [6]. Regular physical exercise is associated with healthy aging and reduced risk of age-related diseases, such as cancer. A good preclinical model to test rapamycin treatment in combination with wheel running would be in mice with syngeneic breast cancer. CB6F1 (BALB/c x C57BL/6) females at 4 months of age were started on rapamycin medicated chow 14 ppm [4] or control chow (5lG6) in cohorts of 20 each. One week later, 10 mice in each cohort were given access to freely rotating wheels (*Med Associates* ENV-044, Vermont), or to wheels that were locked, as described [6,7]. After 10 days, mice were injected with 1 × 10^4^ 4T1 mammary tumor cells into the 4^th^ mammary fat pad and allowed to continue running for another 17 days. Once mammary tumors were palpable, they were measured blindly every three days with digital calipers. Tumor burden was determined using the standard formula V = 1/2 × (width^2^ × length). Interestingly, mice fed the rapamycin chow and given access to wheel running showed a significant increase in tumor burden compared with mice fed rapamycin chow with locked running wheels (P = 0.017) (Figure 1). Individually, neither rapamycin nor wheel running had any anti-tumor effect. This preliminary observation indicates that further studies are needed to determine the adverse effects of combining rapamycin and wheel running in young mice with an aggressive from of breast cancer. Not only does this have implications for young breast cancer patients, but suggests that combining rapamycin and exercise as an anti-aging strategy at a young age might be contraindicated. Although we have not yet identified a specific mechanism, it is possible that rapamycin might be suppressing anti-tumor macrophages, since we have shown that these cells are activated by wheel running in mice [7].

**Figure 1.**
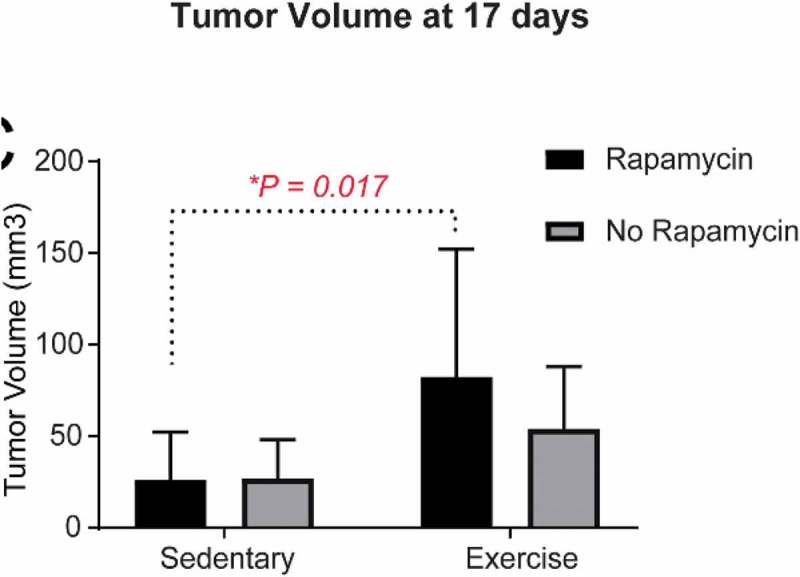
After 17 days of running, mice that were fed a rapamycin diet (14 ppm) had increased tumor burden compared with rapamycin-fed mice that were sedentary (locked running wheels). N = 10 per group, *p = 0.017.
